# Gunshot Wound to the Chest With Retained Epicardial Bullet

**DOI:** 10.7759/cureus.29422

**Published:** 2022-09-21

**Authors:** Toba Bolaji, Abuoma C Ekpendu, Frederick Giberson

**Affiliations:** 1 Surgery, ChristianaCare, Newark, USA; 2 Osteopathic Medicine, Philadelphia College of Osteopathic Medicine, Philadelphia, USA

**Keywords:** cardiac tamponade, trauma surgery, thoracotomy, retained epicardial bullet, gunshot injury to chest

## Abstract

Gunshot wounds remain the most common cause of penetrating injuries in children and adolescents and the second leading cause of death among youth in the United States. Penetrating cardiac injuries carry a significantly increased mortality rate. The extent of damage caused depends on the type of firearm, the bullet used, the velocityand the trajectory. Therefore, rapid diagnosis and treatment is of the utmost importance.

We report a case of a 19-year-old boy who presented to ouremergency department (ED) after sustaining a gunshot wound (GSW) to the right chest. In the ED, the patient was stabilized and a large hematoma was evacuated during a resuscitative thoracotomy. Further thoracotomy in the operating room was done with repairs of the penetrating injuries to the heart and lungs. No bullet was identified after careful inspection of the entire chest in the operating room. However, upon further postoperative imaging, a bullet was identified on chest X-ray and CT, lodged in the anterior aspect of the subepicardial right ventricular outflow tract. After a complicated hospital course, the patient was discharged by hospital day 30 in a stable condition with outpatient follow-up.

The decision to leave or retrieve a bullet should be made on a case-by-case basis depending on the clinical picture. In this case report, we have shown that leaving the bullet in place with close observation and appropriate imaging is feasible for selected patients.

## Introduction

Gunshot injuries are among the leading causes of morbidity and mortality associated with penetrating injuries and are most common in young men in the United States. Penetrating gunshot wounds to the chest are a constant source of challenge to the trauma team. Despite the advances in medical and surgical management, morbidity and mortality continue to be significant. According to the literature, about 90% of patients with penetrating thoracic injuries die before reaching the hospital [1]. Factors that affect survival include the presence of cardiac tamponade, right ventricular injury, and single versus multiple chamber injury. In a study done by Cambell et al, an analysis of patients who died before reaching the hospital showed cardiac tamponade in 18% of isolated cardiac stab wounds [1]. Cardiac tamponade can be advantageous by allowing time for definitive measures [2]. Delay in operative intervention has been shown to be associated with a high mortality rate. Therefore, immediate diagnosis and surgical intervention are imperative to survival.

## Case presentation

Presentation

This is a 19-year-old boy who presented to the ED as a trauma code after sustaining a gunshot wound to the right chest. The pre-hospital call prior to arrival reported a “central gunshot wound”. The patient was in cardiac arrest and was undergoing cardiopulmonary resuscitation (CPR). Upon arrival, an endotracheal airway was noted to be in place and advanced cardiac life support (ACLS) protocol-driven CPR was underway. The end-tidal carbon dioxide (CO2) was 32 mmHg with chest compressions, and a resuscitative thoracotomy was performed. Upon making an incision into the left chest, no blood was seen in the thoracic cavity. The pericardial sac was then incised, and a large hematoma was noted and evacuated, releasing a cardiac tamponade. Return of circulation was noted with a palpable femoral pulse. The aorta was not cross-clamped due to the risk of cardiac or intrapericardial great vessel injury. A right-sided chest tube and a right-sided peripheral intravenous (IV) line were placed simultaneously. The right-sided chest tube immediately yielded greater than 500 milliliters (mL) of blood.

A brief secondary survey was done, revealing one gunshot wound to the right chest at the mid-axillary line. The massive transfusion protocol (MTP) was activated, and an epinephrine drip was started. Following improved vital signs, the patient was transported directly to the operating room for an exploratory laparotomy.

Operating room

In the operating room, a right anterolateral thoracotomy was performed due to persistent chest tube drainage. Once sufficient filling of the heart was achieved, two penetrating wounds on the anterolateral surface of the right ventricle were noted along with pulsatile extravasation. The defects were successfully repaired with 4’0 prolene pledgeted sutures while avoiding the major coronary arteries. The heart was then carefully inspected, and no bullets were identified. Furthermore, penetrating injuries were noted at the middle and lower lobes of the right lung, which were treated with wedge resections. Anesthesiologists were involved throughout the surgery, and they obtained frequent arterial blood gases (ABG) (Table 1) and thromboelastograms (TEG) (Table 2) to direct the resuscitation of the patient. The patient was profoundly acidotic and coagulopathic and as a result, received a total of 18 units (U) of packed red blood cells, 18 units of frozen fresh plasma, and four packs of platelets during the surgery. After another full inspection of the chest and no sources of bleeding identified, chest tubes were inserted bilaterally. Due to persistent shock and profound coagulopathy, his open chest was covered with a closed-system dressing and the patient was transferred to the surgical intensive care unit for continued resuscitation and supportive management.

**Table 1:** Arterial Blood Gas (ABG) Trend - Day of Presentation Table 1 showing improvement in acidosis over the day of presentation represented by serial ABG monitoring. pH arterial: the pH within arterial blood; PCO_2_  arterial: The partial pressure of carbon dioxide (PCO2) within arterial blood; PO_2_ arterial: The partial pressure of oxygen (PO_2_) within arterial blood; Total CO_2_ arterial: Total carbon dioxide (CO_2_) within arterial blood; HCO_3 _arterial: Bicarbonate (HCO_3_) within arterial blood; Base Excess Arterial: Base excess within arterial blood

Time/Result	09:09	09:27	09:42	10:07	11:21
pH Arterial	7.00	7.00	7.00	7.04	7.36
PCO_2_ Arterial	43.1	93.7	73.1	63.3	29.0
PO_2_ Arterial	96	144	150	157	149
Total Co_2_ Arterial	-	-	-	19.0	17.0
HCO_3_	-	-	-	17.2	16.2
Base Excess Arterial	-	-	-	-13.0	-9.0

**Table 2:** Thromboelastograms (TEG) Trend - Day of Presentation Table 2 showing improvement of coagulopathy over the day of presentation represented by serial thromboelastograms (TEG) ACT = Activated clotting time (ACT); R = Reaction time; K = Kinetics; Angle = Alpha angle; MA = Maximum amplitude; LY30 = clot lysis at 30 minutes after maximum strength of clot

Time/Result	09:24	10:02	11:17	15:55
Rapid ACT	308	183	113	
Rapid R	2.8	1.4	0.7	
Rapid K	Undef	6.5	3.6	
Rapid Angle	12.5	37.8	57.9	
Rapid MA	4.6	35.2	44.6	
Rapid LY30	92.9	0.0	0.0	
Kaolin R				5.0
Kaolin K				1.8
Kaolin Angle				65.6
Kaolin MA				59.1
Kaolin LY30				0.0

Post-operative surgical ICU management

**Figure 1:**
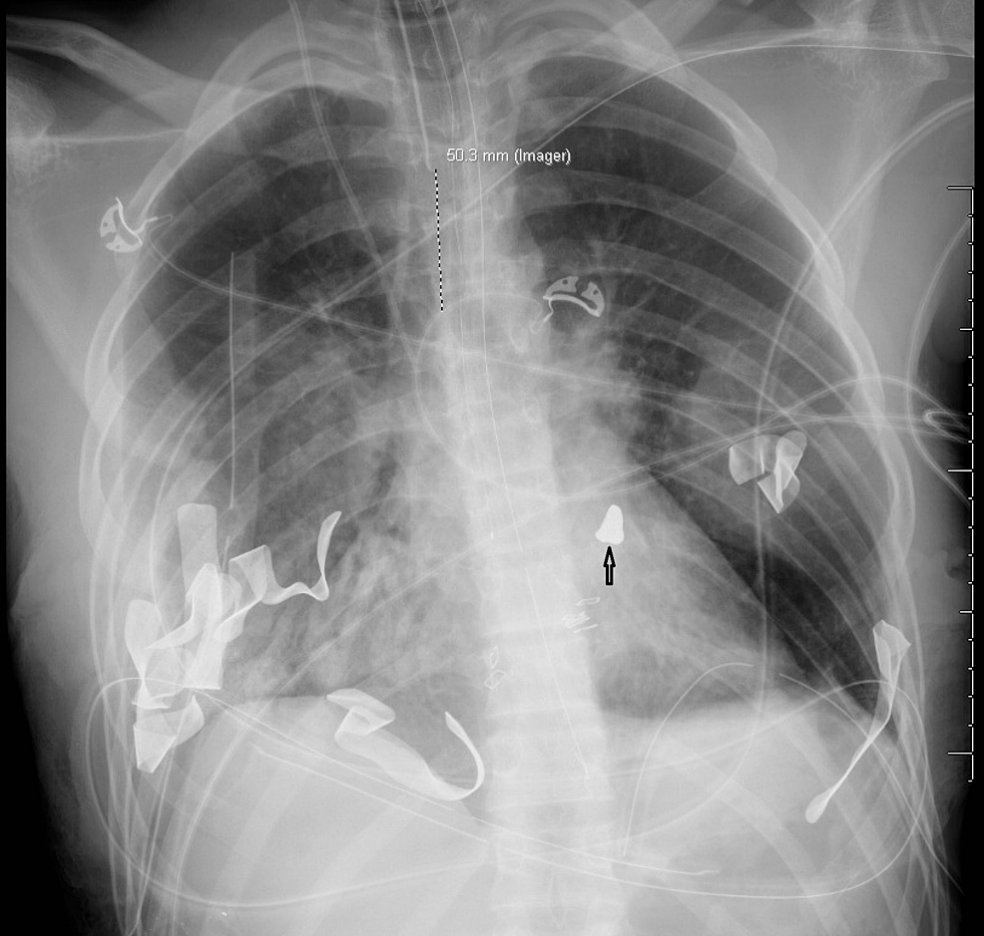
Chest X-ray (CXR) Obtained on Post-Operative Day 1 Figure 1 shows Chest X-ray (CXR) obtained on post-operative Day 1, revealing intrathoracic packing and missile (arrow) within the cardiac shadow.

**Figure 2:**
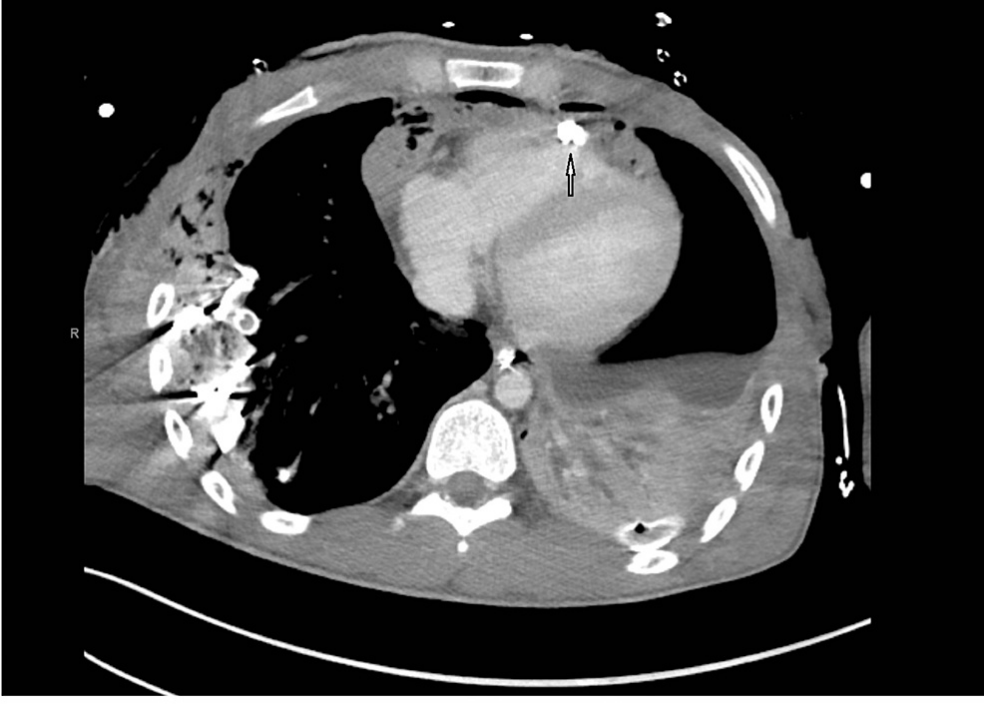
Computed Tomography (CT) Chest Obtained on Post-Operative Day 1 Figure 2 shows Computed tomography (CT) chest obtained on post-operative day 1, revealing missile (see arrow) lodged in the epicardial wall of the right ventricular outflow tract.

Post-operatively, the patient was resuscitated, and his coagulopathy was corrected. Bilateral chest tube outputs decreased substantially over the first operative day. An anteroposterior and lateral chest X-ray was performed, which revealed a bullet projecting within the cardiac shadow (Figure 1). The patient then underwent a transesophageal echocardiogram (TEE), which showed a normal cardiac function. However, the bullet was not visualized on TEE. A computed tomography (CT) scan of his chest demonstrated a foreign body consistent with a bullet lodged in the anterior aspect of the subepicardial right ventricular outflow tract (Figure 2). On post-trauma day 2, the patient underwent chest closure with the removal of surgical packing. During the surgery, the bullet was palpated within the epicardium along the right ventricular outflow tract. In collaboration with the cardiac surgeon consultant, a decision was made to leave the bullet in place since any attempt at removal would have required the patient to be placed on cardiopulmonary bypass, thereby increasing the stress on the patient.

His postoperative hospital course was complicated by acute respiratory distress syndrome (ARDS). He was treated with chest physiotherapy, inhaled nitric oxide and intermittent prone positioning. Unfortunately, he was unable to be weaned from the ventilator, which warranted a tracheostomy placement on hospital day 13. Subsequently, he improved and was weaned to trach collar and decannulated on hospital day 22. Prior to his discharge, a repeat transthoracic echocardiogram was performed, showing adequate cardiac function and the bullet in a stable position. The patient was discharged on hospital day 30 in stable condition with scheduled outpatient follow-up with the trauma and cardiac surgery teams.

Follow-up

The patient was seen within two weeks of discharge by the trauma and cardiac surgery teams for routine follow-up. He was noted to be progressing without issues and his wounds were all healed.

## Discussion

Penetrating cardiac injuries present a challenging circumstance for the emergency/trauma physician. About 90% of patients with penetrating cardiac injury to the heart do not reach the hospital [1]. Similarly, the pre-hospital mortality rate of patients with gunshot wounds to the heart approaches 90% [3]. Consistent with our patient, within the group of people who do survive, cardiac tamponade physiology is usually found. Given the limited capacitance of the pericardial sac and its ability to hold only up to 250 mL of blood acutely, direct compression of the heart by the expanding cardiac tamponade limits cardiac filling and decreases cardiac output and soon after, cardiac arrest ensues. Luckily, the tamponade effect can delay massive hemorrhage from the cardiac injury, allowing time for definitive measures [2].

In this patient population, prompt resuscitation and intervention is a must and seconds make the difference between life and death. Resuscitative thoracotomy is performed in patients with blunt or penetrating thoracoabdominal injuries who present in cardiac arrest (depending on the length of time without signs of life) or progress to cardiac arrest shortly after presentation. The goal of this intervention is to restore cardiac output to the core organs and control any devastating hemorrhage [4]. In 2012, the Western Trauma Association published guidelines which defined the appropriate role of resuscitative thoracotomy in trauma patients. This observational study showed the success of thoracotomy for all patients with penetrating wounds to approach 15% [5]. More recently in 2015, the Eastern Association for the Surgery of Trauma published practice guidelines for the use of ED thoracotomy. In cases of penetrating thoracic trauma, overall survival approached 21% in those with signs of life. Overall survival was 8% in patients without signs of life on presentation [6].

The location of retained thoracic projectiles is a key factor in determining the management plan. When associated with direct cardiac injury, projectiles can be found within the heart chambers, partially or totally embedded in the myocardium, within the pericardial cavity or lodged in proximity to the great vessels [7]. Alternatively, projectiles can be carried to the heart through venous channels and remain free in the right heart chambers, or become embedded within the right ventricular trabeculae, or pulmonary arterial tree.

Immediate complications include cardiac tamponade, hemothorax, valvular insufficiencies, and intracardiac shunt. Late complications include acute myocardial infarction, systemic or pulmonary embolization, pericarditis, endocarditis, ventricular aneurysms, cardiac necrosis and conduction abnormalities.

Proper localization of the object is paramount to determining the appropriate management approach. Work-up should begin with advanced trauma life support (ATLS) protocols. If the patient remains stable, further imaging should then be pursued to determine the specific location of the bullet to tailor management [8]. Computed tomography remains the preferred imaging modality in hemodynamically stable patients with thoracic trauma, allowing for fast and accurate examination of the entire chest [9].

The management of penetrating cardiac injuries involving retained bullets is controversial. The three management approaches include observation, open retrieval, and endovascular extraction depending on the location of the bullet. In 2018, Yoon et al proposed the following algorithm regarding the management of a bullet to the heart and the great vessels: symptomatic patients should undergo retrieval using endovascular extraction as the first line. Failure of endovascular extraction should warrant consideration for open retrieval. Patients with a history of atrial septal defect, ventricular septal defect, or patent foramen ovale should have an open retrieval due to concerns for possible stroke or distal emboli. Asymptomatic patients can be observed and if symptoms develop, retrieval can be pursued [10]. Factors to consider in observation and serial imaging include a smooth appearing bullet, <5 mm in diameter, firmly lodged, uncontaminated, in a hemodynamically stable patient without evidence of arrhythmias or valvular dysfunction. In 1990, Symbas et al reviewed reported cases in the English literature from 1940 to 1988 and their own experiences from 1968 to 1988, arriving at the following suggestions: retained projectiles within the pericardial space or completely embedded in the pericardium or myocardium are generally well-tolerated and may be left in place. Shrapnel should be removed if it is not completely embedded. Intracavitary missiles (bullets, pellets), or those partially embedded in the left myocardium should be removed. However, intracavitary missiles in the right side of the heart (excluding embolized missiles from the intestinal viscus), may be observed while anticipating their embolization to the pulmonary artery, from which they can be retrieved. Missiles located next to a coronary artery should be removed. In the series by Symbas et al, the two patients who had bullets that were not removed from the pericardium had no symptoms or complications [11].

The decision of whether to leave a projectile in the heart or retrieve it is not straightforward. It should be individualized on a case-by-case basis and adapted to the level of experience and resources available while involving the patient in the decision-making process when feasible.

In the case of our patient, the bullet was lodged in the anterior aspect of the subepicardial right ventricular outflow tract. Removing it would have required an open approach, with the patient having to be placed on bypass, which would have added more stress to the patient. Moreover, given its location, the chances of embolization were low and the patient remained asymptomatic after his recovery.

## Conclusions

Penetrating thoracic trauma, specifically cardiac injuries, present a challenging circumstance for the ED/Trauma physician. For the minority of patients who survive long enough to present to the hospital, prompt resuscitation and quite often surgical intervention for definitive hemorrhagic control are required. The management of a lodged/retained bullet is a challenging one that is dependent on location and hemodynamic stability, level of experience of the surgeon, and available resources. Diagnostic imaging, specifically echocardiogram and computed tomography, are useful in localizing the object. The morphology of the object and its location within the heart are necessary factors to consider. Although gunshot wounds to the heart are a common occurrence, there is limited literature reporting observation as the chosen management option. In this report, we have shown that leaving the bullet in place with close observation and appropriate imaging is feasible for selected patients.
